# Neurological Manifestations of HEV Infection: A Rare Phenomenon or an Underrecognized Reality?

**DOI:** 10.1002/brb3.70585

**Published:** 2025-06-18

**Authors:** Maider Iza, Daniel Ramos, Arnau Llauradó, Juan Luis Restrepo‐Vera, Mercedes Pallero, Galo Granados, Jose Alemany, Javier Sotoca, Júlia Sampol, Sergi Martí, Daniel Sanchez‐Tejerina, Maria Salvadó, Raul Juntas

**Affiliations:** ^1^ Neurology Department Vall d´Hebron University Hospital Barcelona Spain; ^2^ Pneumology Department Vall d´Hebron University Hospital Barcelona Spain; ^3^ Neuromuscular Pathology Department Vall d´Hebron University Hospital Barcelona Spain

**Keywords:** hepatitis E virus, immunoglobulins, meningoradiculitis, neuralgic amyotrophy, neurologic manifestations

## Abstract

**Aim:**

This study aimed to describe neurological manifestations secondary to hepatitis E virus (HEV) through the description of two clinical cases.

**Methods:**

Two different cases of neuralgic amyotrophy and meningoradiculitis are evaluated in the emergency department of a tertiary referral hospital in 2024.

**Results:**

*Case 1*: A 43‐year‐old male presented to the emergency department with proximal weakness and pain in the right upper extremity associated with acute onset of orthopnea. Laboratory tests revealed elevated AST/ALT levels (184/1164 IU/L) and positive HEV IgM and IgG, with detectable serum HEV viral load. Cerebrospinal fluid (CSF) was negative for HEV RNA. A significant decrease in forced vital capacity was observed on transition from the upright to the supine position. Electromyography showed severe bilateral phrenic nerve involvement. The diagnosis of neuralgic amyotrophy with diaphragmatic paralysis secondary to HEV was made. The patient was treated with intravenous immunoglobulins and noninvasive ventilation with partial improvement*. Case 2*: A 37‐year‐old male presented to the emergency department with paresthesias and weakness, initially affecting the distal upper and lower extremities and progressing proximally. Laboratory tests showed elevated AST/ALT levels (238/626 IU/L), positive HEV IgM and IgG, and a detectable HEV viral load in serum. HEV RNA was also detected in the CSF. Neurophysiological findings were normal. The patient was diagnosed with acute meningoradiculitis secondary to HEV. Treatment with intravenous immunoglobulins led to complete resolution of symptoms.

**Conclusions:**

In cases of acute neurological symptoms and liver dysfunction, HEV should be considered as a potential causative agent.

## Graphical abstract

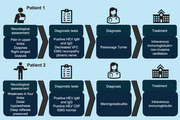

## 1 Introduction

Hepatitis E virus (HEV) is a significant contributor to liver disease worldwide, accounting for up to 70% of adult sporadic hepatitis cases in endemic regions (Iqbal et al. 2023). In developed countries, it is primarily transmitted to humans through the consumption of contaminated meat or water contaminated with fecal matter (Goel and Aggarwal 2017; Kenney and Meng 2019; Aslan and Balaban 2020).

Recent studies have shed light on the extrahepatic manifestations of HEV infection, particularly its neurological complications such as neuralgic amyotrophy (NA) or Parsonage–Turner syndrome (PTS), Guillain–Barré syndrome (GBS), encephalitis/myelitis, mononeuritis multiplex or meningoradiculitis (Ripellino et al. 2018; Woolson et al. 2014; Dalton et al. 2016; Jha et al. 2021). The current recommended approach for neurological complications is to modulate the immune response that typically involves using intravenous immunoglobulin (IVIG) administered over 5 days, with a dosage of 2 g/kg (Abravanel et al. 2018). There is also some evidence supporting the use of prednisolone with a gradual tapering off (Ripellino et al. 2019; Van Eijk et al. 2009).

## 2 Patients and Methods

A comprehensive description is provided for two cases of patients admitted to the University Hospital of Vall d'Hebron, Barcelona, Spain. Data were collected from the medical histories of the two patients after prior informed consent.

### 2.1 Case Report 1

**FIGURE 1:**
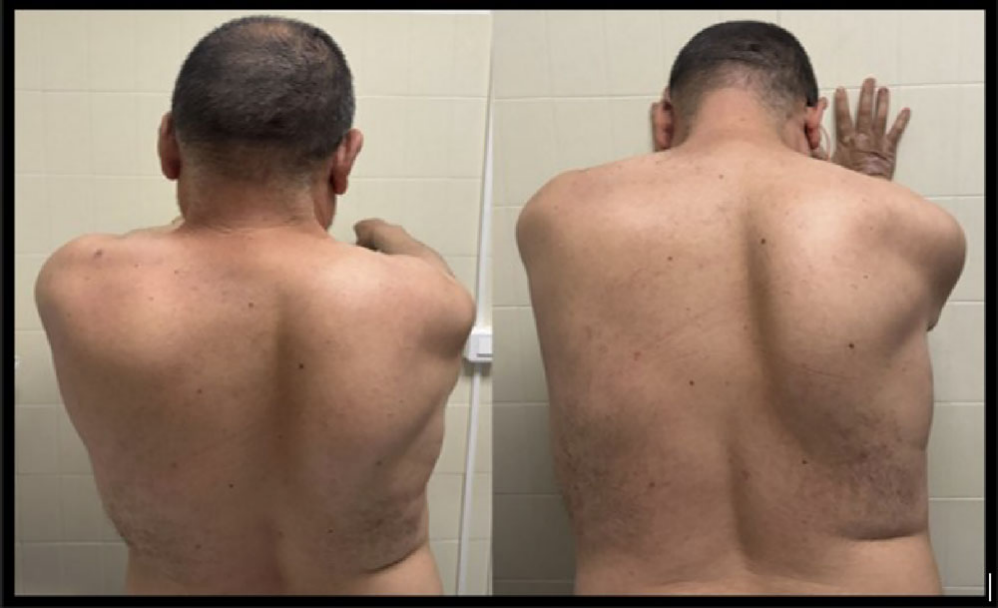
Physical examination of patient #1 showing right winged scapula, amyotrophy in supraspinatus and infraspinatus fossae, and abduction impairment.

**FIGURE 2:**
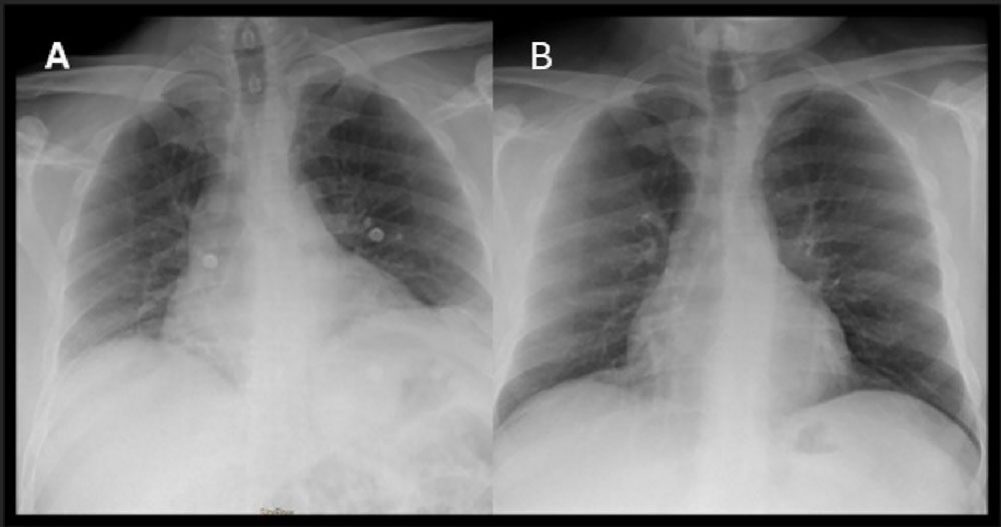
Chest x‐ray performed in patient #1. (A) Chest x‐ray: April 6, 2024: Mild elevation of both diaphragms. (B) Chest x‐ray: August 3, 2024: No elevation can be observed in diaphragms.

A 43‐year‐old man with a medical history of type 2 diabetes, hypertriglyceridemia, psoriasis, and schizophrenia consulted at the emergency department on March 24 due to pain in both upper limbs and right brachial weakness, associated with sudden dyspnea and orthopnea. The patient did admit to having previously eaten undercooked pork meat. The physical examination showed no indications of liver failure. He had slight weakness of the right deltoid muscle as well as right right‐winged scapula (Figure 1). Bilateral diaphragmatic elevation was observed on the chest x‐ray (Figure 2). The blood analysis depicted hepatic cytolysis and mild cholestasis (Table 1). EBV, HAV, HBV, HCV, HIV, CMV, *Coxiella burnetii*, and *Toxoplasma gondii* were excluded. HEV serology resulted in positive IgM and HEV RNA positive (83,000 International Unit [IU]/mL). We did not perform HEV genotype sequencing. CSF analysis showed mild hyperproteinorrachia of 74 mg/dL and no pleocytosis. HEV RNA analysis yielded a negative result in the CSF. An electrophysiological study revealed impairment of the right long thoracic nerve and severe neuropathy of both phrenic nerves, characterized by significantly reduced amplitudes and prolonged distal latency. Respiratory function tests revealed a 49.8% forced vital capacity decrease from upright to supine position (from 2.66 to 1.34 L) (Table 1).

**TABLE 1:** Demographic, clinical, biochemical, virologic, CSF analysis, and neurophysiologial data of the two patients with HEV‐associated neurological manifestations.

	Patient 1	Patient 2
Demographic features		
Age at onset	43 years	37 years
Sex	Male	Male
Immunocompetent	Yes	Yes
Clinical symptoms and course	Pain and weakness in right arm. Dyspnea and orthopnea	Paresthesia and weakness in upper and lower limbs
Antecedent events	Ingestion of undercooked meat	Ingestion of undercooked meat
Clinical phenotype	Bilateral, right > left	Bilateral, right upper limb > left upper limb Left lower limb > right lower limb
Clinical course and outcome at outpatient basis	No longer pain, residual weakness and dyspnea	Full recovery
Weakest muscles at outpatient basis	Deltoid muscle, right > left	None
mRS scale at onset	2	1
mRS scale at outpatient basis	1	0
Biochemical and virologic studies		
Timing (days after symptom onset)	1	4
AST (8–34 IU/L)	184	238
ALT (10–49 IU/L) FA (46–116 IU/L) GGT (7–73 IU/L)	1164 221 223	626 181 399
Bilirubin (0.30–1.20 mg/dL)	0.33	1.48
Autoimmune study	ANA and anti‐SSA (Ro60) positive	Negative
Anti‐HEV	IgM and IgG positive	IgM and IgG positive
Initial HEV viral load, log IU/mL	83,000 IU/mL	22,000 IU/mL
HEV viral load, log IU/mL at outpatient basis	Negative	86 IU/mL
CSF analysis		
Basic parameters	No pleocytosis, mild hyperproteinorrachia of 74 mg/dL	Pleocytosis of 55 cells/µL with 100% lymphocytes, mild hyperproteinorrachia of 113 mg/dL
HEV RNA	Positive	Positive
Neurophysiological study		
Pattern	Demyelinating	Not altered
Most affected nerves	Right long thoracic nerve and bilateral phrenic nerves	None

Abbreviations: ALT, alanine aminotransferase; AST, aspartate aminotransferase; D, deltoid; HEV, hepatitis E virus; Ig, immunoglobulin; mRS, modified Rankin scale; NA, neuralgic amyotrophy.

Thus, the diagnosis of NA with diaphragmatic paralysis secondary to HEV was made. Treatment consisted of IVIG at a dose of 0.4 g/kg/day for 5 days and noninvasive mechanical ventilation. Corticosteroid therapy was not applied due to active viral replication. Ribavirin was also not administered.

During his hospital stay, the HEV viral load in the blood decreased progressively to undetectable levels. Liver function parameters also normalized. In addition, the patient experienced neurological improvement with increased strength and decreased pain. Given the patient's favorable progression, he was discharged from the hospital on April 16.

Three months later, at the time of the last assessment, he still experienced a slight difficulty in arm abduction. Additionally, he required the use of noninvasive mechanical ventilation during sleep and naps.

### 2.2 Case Report 2

A 37‐year‐old male with no significant past medical history presented to the emergency department on March 21 with a 10‐day history of tingling and weakness that began in the hands and progressed to the arms, followed by similar symptoms in the lower extremities, beginning in the feet and progressing proximally to the thighs. The patient acknowledged having previously consumed undercooked pulled pork. Physical examination did not show any signs of liver failure. Two days prior to the onset of these symptoms, he had experienced a self‐limited episode of diarrhea.

During physical examination, the patient had a slight distal weakness in flexors and extensors of the wrist and in the interosseus muscles of the right upper limb and a slight proximal weakness in quadriceps and hamstring of the lowers limbs. The reflexes were absent. Additionally, the patient exhibited hypoesthesia in the distal regions of both the upper and lower extremities.

The blood analysis showed hepatic cytolysis and cholestasis. Blood tests rendered a positive result for HEV IgM antibodies and a positive HEV PCR (22,000 IU/mL). We did not carry out HEV genotype sequencing. Serologic analysis showed negative results for HIV, HAV, HBV, and HCV.

A posterior lumbar puncture and CSF analysis revealed pleocytosis of 55 cells/µL with 100% lymphocytes and mild hyperproteinorrachia of 113 mg/dL. Additionally, the CSF was positive for HEV RNA, with a viral load of 250 IU/mL. An electrophysiological study was also performed that exhibited normal sensoy and motor nerve conduction, along with normal F responses and somatosensory evoked potentials (SSEP) (Table 1).

The cerebral and spinal MRI was not performed due to the subsequent rapid clinical recovery of the patient, although the findings of areflexia and distal sensory deficit led us to suspect a probable polyradiculopathy. Therefore, a diagnosis of meningoradiculitis due to HEV was made. IVIG (0.4 g/kg/day for 5 days) was initiated. He did not receive corticosteroid therapy due to ongoing viral replication; ribavirin was neither associated. Five days after initiating treatment, he reported an almost complete resolution of symptoms. During his hospital stay, the levels of transaminases were gradually normalized, and viral load of HEV in plasma was also reduced (viral load of 2100 IU/mL on March 25). Thus, the patient was discharged on March 26.

In the follow‐up visit 2 weeks after hospital discharge, he had a completely normal neurological examination. The blood analysis showed almost normal levels of AST and ALT at 44 IU/L and 97 IU/L, respectively. HEV replication was also reduced, with a viral load of 86 IU/mL.

## 3 Discussion

Neurological complications have been previously reported in individuals infected with HEV, including GBS, NA, encephalitis, myelitis, and meningoradiculitis (Dalton et al. 2016). Recent research has also explored the relationship between HEV exposure and chronic inflammatory demyelinating polyneuropathy (CIDP) (Pischke et al. 2024).

In our two patients, the liver function abnormalities were mild and neither had clinical jaundice. Both patients developed HEV‐associated neurologic manifestations following an acute onset. This highlights the importance of considering viral hepatitis as a diagnostic possibility even when clinical or biochemical signs are subtle, a point that has been highlighted in previous studies (Mclean et al. 2017).

The patient affected by NA had bilateral brachial plexus involvement leading to secondary diaphragmatic paralysis. This finding is consistent with previous reports (Van Eijk et al. 2017; Scanvion et al. 2017). Electrophysiological examination revealed a patchy multineuropathy previously described in NA and HEV (Velay et al. 2017). Although phrenic nerve involvement has been noted (Scanvion et al. 2017), it is crucial to emphasize the severe and acute nature of the involvement in this case, which required the implementation of noninvasive mechanical ventilation.

The patient with meningoradiculitis presented with paresthesias and weakness in the four limbs. Other studies have also reported an association between HEV and meningoradiculitis (Perrin et al. 2015; Kamar et al. 2010). It is important to highlight in our patient the clear alteration of CSF characterized by lymphocytosis and a high level of protein, despite the EMG results being entirely normal. It is also important to highlight that due to the significant clinical improvement exhibited by the patient, a cerebral or spinal MRI was not conducted. This decision could be considered a potential limitation in this case.

However, the first patient had a positive HEV viral load in the CSF, we did not observe this in the second patient. This indicates the two hypotheses: one could be that HEV has a preference for neuronal tissue due to neurotropic variants, and the other one would be that it is an immune‐mediated entity, resulting in cross‐reactions between viral epitopes and self‐antigens (Perrin et al. 2015; Abravanel et al. 2018; Fousekis et al. 2020). Unfortunately, there was no evaluation conducted on the intrathecal synthesis of anti‐HEV IgM in the CSF.

The first patient did show seroconversion after 1 month of clinical onset, whereas the second patient still showed positivity for HEV RNA after 2 weeks of clinical onset. This is due to the fact that HEV RNA is detected in the bloodstream for approximately only 4 weeks, while IgM+ can be detected for a longer period of 6–9 months (Kamar et al. 2017; Huang et al. 2010). This also means that patients who are referred late to the neurologist may present a negative HEV PCR and it should be taken into account when assessing these patients.

Both the patients were treated with IVIG. There is not much evidence regarding immunomodulatory treatment in patients with HEV and neurological complications, although some reports have been published where an improvement in neurological symptoms has been observed (Perrin et al. 2015; Abravanel et al. 2018; Silva et al. 2016).

In conclusion, this study delineates two cases that support the causal relationship between HEV and neurological complications. Clinicians should have a low threshold for testing HEV in patients with unexplained neurological symptoms and abnormal liver function test results. Early diagnosis and immunomodulatory treatment may lead to a better prognosis, reducing the chances of long‐term neurological complications.

## Author Contributions

**Maider Iza**: conceptualization, data curation, formal analysis. **Daniel Ramos**: data curation, formal analysis. **Arnau Llauradó**: conceptualization, data curation, formal analysis, investigation, project administration. **Juan Luis Restrepo‐Vera**: methodology, project administration. **Mercedes Pallero**: resources, supervision. **Galo Granados**: resources, supervision. **Jose Alemany**: methodology, project administration. **Javier Sotoca**: investigation, methodology, project administration. **Júlia Sampol**: resources, supervision. **Sergi Martí**: resources, validation. **Daniel Sanchez‐Tejerina**: conceptualization, data curation, investigation, methodology, project administration. **Maria Salvadó**: conceptualization, data curation, formal analysis, investigation, methodology, project administration. **Raul Juntas**: conceptualization, data curation, formal analysis, investigation, methodology, project administration.

## Ethics Statement

We confirm that we have read the journal's position on issues involved in ethical publication and affirm that this report is consistent with those guidelines.

## Consent

Patients consented for the publication of details.

## Conflicts of Interest

The authors declare no conflicts of interest.

## Peer Review

The peer review history for this article is available at https://publons.com/publon/10.1002/brb3.70585

## Data Availability

The authors confirm that the data supporting the findings of this study are available within the article.
